# Ellagic acid alleviates motor, cognitive and hippocampal electrical activity deficits in the male rats with 2-vessel occlusion cerebral ischemia/reperfusion

**DOI:** 10.22038/AJP.2023.22787

**Published:** 2023

**Authors:** Khadijeh Hassonizadeh Falahieh, Alireza Sarkaki, Mohammadamin Edalatmanesh, Mohammad Kazem Gharib Naseri, Yaghoob Farbood

**Affiliations:** 1 *Department of Physiology, College of Sciences, Science and Research Branch, Islamic Azad University, Fars, Iran*; 2 *Department of Physiology, College of Sciences, Shiraz Branch, Islamic Azad University, Shiraz, Iran*; 3 *Medicinal Plants Research Center, Ahvaz Jundishapur University of Medical Science, Ahvaz, Iran*; 4 *Persian Gulf Physiology Research Center, Medical Basic Sciences Research Institute, Ahvaz Jundishapur University of Medical Science, Ahvaz, Iran*; 5 *Department of Physiology, Medicine Faculty, Ahvaz Jundishapur University of Medical Science, Ahvaz, Iran*

**Keywords:** Cerebral Ischemia/Reperfusion, Ellagic acid, Motor coordination, Cognition, Hippocampal local EEG, Oxidative stress

## Abstract

**Objective:**

Cerebral ischemia/reperfusion (I/R) has been known as a major cause of inability and mortality worldwide. Ellagic acid (EA) has many pharmacological effects including antioxidant, antithrombotic and neurorestoration activities. The aim of this study was evaluation of the effects of EA on motor and cognitive behaviors, hippocampal local field potential (LFP), brain oxidative stress in male rats with cerebral 2-vessel occlusion ischemia/reperfusion (2VO I/R).

**Materials and Methods:**

Forty-eight male Wistar rats (250-300 g) were assigned into six groups. 1) The Sham: rats were treated with DMSO10%/normal saline as solvent of EA 3 times daily for 1 week; 2) I/R+Veh; I/R rats received vehicle; 3-5) EA-treated groups: I/R rats received 50, 75, or 100 mg/kg EA; and 6) Cont+EA100: intact rats received EA. The cerebral 2VO I/R was made by the bilateral common carotid arteries closing for 20 min followed by reperfusion. The behavioral tests and hippocampal LFP recording were performed after treatment with EA. The oxidative stress parameters were assayed by special ELISA kits.

**Results:**

Cerebral 2VO I/R significantly decreased motor coordination, memory and hippocampal LFP and significantly increased oxidative stress. Treatment with EA improved all I/R complications.

**Conclusion:**

The current findings showed that treatment of I/R rats with EA could reverse cognitive and motor functions, and improve the LFP and oxidative stress markers. So, effects of EA on cognitive and motor function may at least in part, be due to its antioxidative actions.

## Introduction

Cerebral ischemia has been known as a major reason of inability and mortality worldwide (Flynn et al., 2008). The main harm from ischemia can be due to inflammation, oxidative stress or apoptosis (Sánchez, 2013 ▶). Oxidative stress is highly involved in tissue damage caused by ischemia/reperfusion (I/R) by increasing the level of reactive oxygen species (ROS) resulting in lipid peroxidation (LPO) and DNA damage (Nita et al., 2001 ▶). The pathophysiology of the stroke is very complicated and involves numerous processes, such as reduced energy, excitotoxicity, oxidative stress, blood-brain barrier (BBB) interruption, neuro-inflammation, necrosis or apoptosis, etc. (Guo et al., 2013 ▶). A large amount of ROS is produced during an acute ischemic stroke, and oxidative damage is a vital mediator of tissue injury in this disease (Lakhan et al., 2009 ▶). I/R injury leads to localized and systemic inflammatory responses that produce oxidant, complement activation, endothelial leukocyte adhesion, transendothelial leukocyte migration, leukocyte-platelet aggregates, increased microvascular permeability, and reduced endothelium-dependent relaxation (Carden and Granger, 2000 ▶). ROS caused to regulate survival/death in the cell by activating different signaling cell pathways, such as p38, c-Jun N-terminal kinases, nuclear factor-kappa B (NF-kB) pathway, Janus kinase/signal transduction and JAK/STAT transcription activators (Hou et al., 2016 ▶). Cerebral ischemia accelerates the degradation of energy storage, which results in a complicated cascade of cellular processes, like cellular depolarization and Ca^+2^ infiltration, leading to the death of excitotoxic cells (Harukuni and Bhardwaj, 2006 ▶). Oxidative stress leads to cell loss through DNA damage, cell membrane lipid peroxidation, and changing the protein structure and performance (Sun et al., 2018 ▶). Cerebral ischemia causes neurological disorders, like motor and speech problems as well as neurological defects such as cognitive and apraxia disorders, spatial learning and memory impairment (Hosseinzadeh et al., 2007 ▶). In addition, patients who live for more than six months after stroke have some complications such as paralysis of a part of the body, memory impairment, and thinking, talking and moving deficits (West et al., 2008 ▶). It has been shown that among neurons of the brain, pyramidal cells from the CA1 area of the hippocampus are more susceptible to ischemic conditions (Bin et al., 2012 ▶). 

The power of rhythmic slow activity recorded during freely moving behavior and the power of slow delta activity during alert immobility decreased monotonically, with large alterations occurring between post-ischemic days 2 and 4. The changes in spontaneous activity were accompanied by a decrease and eventual disappearance of the Schaffer collateral evoked responses in CA1. Perforant path streams were efficient in activating the granule cells following ischemia compared to baseline levels. Neurophysiological signs of spontaneous or evoked neuronal hyperexcitability were not observed at any time point during 8 days after ischemia. Neuronal damage in the hippocampal CA1 region varied from moderate to complete loss of pyramidal cells. In addition, degenerating neurons were also observed in the hilus of the dentate gyrus (Buzsaki et al., 1989 ▶). 

Considerable evidence has shown that a few plant products, medicinal plant extracts, and phytochemicals including polyphenols, terpenes, and flavonoids, exert important antioxidant and anti-inflammatory effects and are effective in several chronic neurodegenerative diseases (Maurya and Rizvi, 2009 ▶; Pandey and Rizvi, 2009 ▶; Perez-Hernandez et al., 2016 ▶). In recent times, one of the polyphenol compounds, ellagic acid (EA), a metabolite of ellagitannin, has received much considerations due to its many physiological and pharmacological actions. Ellagic acid is present in many fruits such as pomegranates, persimmons, and raspberries, among others. Structurally, it has four hydroxyl groups that are responsible for its antioxidant property. The medicinal and pharmacological properties of EA have been evaluated and discussed elsewhere (Rios et al., 2018 ▶).Several studies proposed that EA has many therapeutic profits such as being anti-inflammatory, neuroprotective, hepatoprotective, antidiabetic, and anticancer, and beneficial against cardiovascular disease (BenSaad et al., 2017 ▶; Ceci et al., 2018 ▶; de Oliveira, 2016 ▶). EA is well recognized for scavenging free radicals, such as antioxidant vitamins C and E. Alongside its antioxidant role, EA has been shown to exert potent anti-inflammatory activities by inhibiting pro-inflammatory cytokines (Hassanzadeh, 2018 ▶).

In the current research, the impacts of EA on motor and cognitive functions, hippocampal local field potential (LFP) recording, and brain tissue oxidative stress in male rats with brain I/R, were studied.

## Materials and Methods

**Animals**

In this experimental study, 48 male Wistar rats (250-300 g) were obtained from the Ahvaz Jundishapur University of Medical Sciences (Ahvaz, Iran) and assigned randomly in 6 groups. Animals were housed in the opaque polycarbonate cages, under the standard conditions of the 12-hours dark-light cycle (07:00 am-07:00 pm), under controlled conditions regarding heat (20 to 22°C) and humidity (55-60%), and were fed with an ordinary diet. All experiments were done in agreement with Ethics guides approved by Ethics Committee of Islamic Azad University of Shiraz, Fars (IR-iaushiraz-95-11-758).

**Experimental groups**

**Figure 1:**
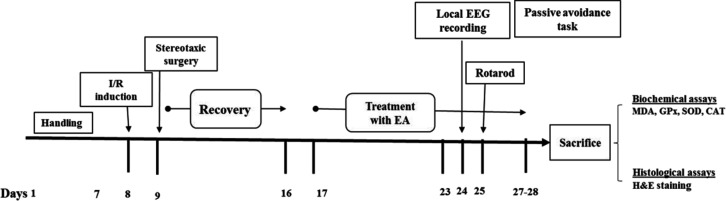
Schematic diagram of time line and experimental design. I/R; ischemia/reperfusion, EEG: Electroencephalogram, EA: Ellagic acid, H&E: Haemotoxylin and Eosin

1) Sham: rats subjected to the surgery without the 2VO I/R were treated with DMSO10%/ saline as solvent of EA (5 ml/kg, gavage) 3 times daily to the end of behavioral tests. 2) I/R+Veh: I/R rats received DMSO10%/normal saline three times daily (at 06:00 am, 2:00 pm and 10:00 pm) to the end of behavioral tests. 3-5) EA-treated groups (I/R+EA50, I/R+EA75 and I/R+EA100); I/R rats received 50, 75, or 100 mg/kg EA (Hassonizadeh Falahieh et al., 2020 ▶) three times daily (similar to I/R+Veh group) (to the end of behavioral tests. 6) Cont+EA100: healthy intact rats received 100 mg/kg EA 3 times daily in order to identify the effect of EA in healthy rats with physiological condition. Time line and experimental design of all protocols is shown in Figure 1. 

**2VO cerebral ischemia/reperfusion (I/R) induction**

Surgery for cerebral I/R (2VO) was performed gradually in the anesthetized rat. 

Rats were anesthetized with ketamine/xylazine (90:10 mg/kg, ip). An incision was made through the ventral midline of the neck. This stage guarantees a reversible obstacle of the bilateral common carotid arteries. Both right and left carotid arteries were then exposed and slightly isolated from the vagal nerve. Occluding was done for 20 min by the small vessel clips simultaneously. Then, the clips were detached to allow to re-flow the blood toward the brain. The incision was sutured followed by transferring the rats to their home cages. After the successful induction of cerebral I/R, symptoms, including loss of consciousness and righting reflex were assessed in the injured animals (Ravindran, 2019 ▶).

**Electrophysiology and behavioral tests**

**Recording and analysis local field potential (LFP)**

In order to record the local EEG, a pair of small screw and a bipolar stainless steel metal wire electrode were implanted into hippocampal CA1 area of rats with stereotaxic coordination AP; -4.56 mm related to Bregma, ML; 2.2 mm from midline and DV; -3 mm from skull surface (Paxinos and Watson, 2007 ▶). Under anesthesia with ketamine and xylazine (80/5 mg/kg, ip). Local EEG was recorded by a ML135 bio-amplifier 4-Channels Power Lab, Lab Chart software version 7.3, (AD Instruments, Australia) with the amplification of 1 mV, sample recording of 400 Hz, and band pass filtration of 0.3–70 Hz within 5 min (Meier, et al., 2020 ▶). 

**Passive avoidance task (PAT)**

The PAT behavior as an index of memory was evaluated in a shuttle box device (Burj Sanat Azma Co., Tehran, Iran). The device consisted of two boxes made with same dimensions, 14 × 14.5 × 27 cm, as a bright (safe area) and dark (unsafe area) chambers. There was a guillotine door between the two sections. Metal bars (2 mm in diameter) with 10 mm intervals on the dark floor were used to deliver a light electrical shock to the animal's feet. In the current study, the passive avoidance memory was tested as follows: 1). Adaptation and habituation stage: at this stage, each rat was placed into the light section to freely move in order to familiarize with the device spaces for 10 min while the guillotine door was open. 2) Initial latency measuring stage: At this stage, 24 hr after the adaptation, rats were put into the bright chamber and 10 sec later, the guillotine door was upraised and the latency to pass the animal from the bright section into the dark section was recorded, this latency was referred to as "initial latency (IL)". After entering the animal, the guillotine door was pulled down and a light electric shock (1.5 mA, 3 sec) was given to the soles of the feet of the animal. After 2 min, the animal was transferred into its cage. 3) Retention test; this step is the same as the previous one except that it lacked any shock delivery. The rat was placed in the lighted chamber and the guillotine door was opened 10 sec later. At this stage, the time of step-through latency (STL) was recorded as memory retaining. The STL stage refers to the time the animal is present in the lighted chamber before entering the dark chamber after opening the guillotine door. At this stage, the maximum time is 300 sec as the cutoff time (Mohammadi, 2017 ▶; Tamburella, et al., 2012 ▶). 

**Measurement of motor activity**

Balance maintenance and resistance for motor coordination in rats were measured using a rotarod apparatus (Burj Sanat Azma Co., Tehran, Iran). The rats from different groups were placed in the rotarod apparatus (graduated rotating from 5 to 40 rpm/12 min), equipped with a rolling belt which adjusted the speed. The balance maintenance duration as seconds on the rotating rod was compared between tested groups. The balance maintenance duration for each rat was measured three times with 45 min intervals, and the mean values were calculated (Crawley, 2003 ▶). 

**Biochemical study**

**Measuring oxidative stress in hippocampal tissue**

At the end of the experiments, the animals were sacrificed under deep and irreversible anesthesia with an overdose of sodium thiopental (100 mg/kg, i.p.) and their brains were removed from the skull in less than 2 min, followed by rinse with saline. The brains were carefully weighed and kept in a special container at -80°C freezer until the test was carried out. On the test day, the samples were homogenized in a phosphate buffer saline (PBS) and then centrifuged at -4°C at 3000 g for 15 min. Then, the supernatant was used to measure the concentration of oxidants by special kits.

**Catalase (CAT) assay**

The Goth’s colorimetric method (1991) using ammonium molybdate and hydrogen peroxide (H_2_O_2_) was used to evaluate CAT activity. It was measured using hydrogen peroxide. The reaction was interrupted after the addition of ammonium molybdate. The CAT enzyme in the sample is for converting H_2_O_2_, therefore, a yellow complex is formed. Following H_2_O_2 _and ammonium molybdate reaction, optical absorbance at 410 nm was read by microplate reader. The amount of enzyme activity is reported as U/ml (Goth, 1991 ▶). 

**Glutathione peroxidase (GPx) enzyme assay**

The GPx enzyme assay was done by ZellBio Kit (GmbH, Ulm, Germany). The GPx enzyme catalyzes the oxidation reaction of glutathione by cumene hydroperoxide. In the presence of glutathione reductase and nicotinamide adenine dinucleotide phosphate (NADP), oxidized glutathione is changed to reductive glutathione which is associated with the simultaneous oxidation of NADPH to NADP. In this reaction, reduction of optical absorption was measured at 412 nm, and enzyme activity is reported as U/ml (Nagababu, et al., 2010 ▶). 

**Measurement of superoxide dismutase (SOD) activity**

To determine SOD activity, a specific kit (ZellBio GmbH, Ulm, Germany) was used, based on the manufacturer’s protocol by conversion of superoxide to hydrogen peroxide and oxygen. One unit of SOD activity is equivalent to the amount of protein that accelerates the decomposition of 1 µmoL of superoxide (O^2-^) into hydrogen peroxide and oxygen during one minute. According to the manufacturer’s instructions, the required amounts of homogenized tissue solutions were incubated with the reagents in a microplate for 20 min at 37°C. Optical absorption was read at 420 nm, using an ELISA microplate. The level of enzyme activity is expressed as U/ml (Nagababu et al., 2010 ▶). 

**Measurement of malondialdehyde (MDA) level**

Thiobarbituric acid (TBA) was used to measure the tissue concentration of MDA. MDA reacts with TBA to produce a red colored complex that has peak absorbance at 532 nm. To measure the concentration of MDA in the ischemic brain tissue 3 ml phosphoric acid (1%) and 1 ml TBA (0.6%) were added to 0.5 ml of homogenate in a centrifuge tube and the mixture was heated for 45 min in a boiling water bath. After cooling, 4 ml n-butanol was added to the mixture and vortex-mixed for 1 min followed by centrifugation at 2000 g for 20 min. The colored layer was transferred to a fresh tube and its absorbance was measured at 532 nm. TBARS levels were determined using 1,1,3,3-tetramethoxypropane as a standard. The standard curve of MDA was constructed over the concentration range of 0–20 µM (Mansouri et al., 2013 ▶). 

**Histological study**

At the end of the experiments to confirm the neuronal survival in the cortex and hippocampus, animals were deeply anesthetized with an overdose of sodium thiopental (100 mg/kg, ip). The brains were removed carefully for histopathological examinations. The samples were fixed in 10% buffered formalin. The tissues were implanted in paraffin to cut into slices, then stained with hematoxylin and eosin (H&E) and examined under light microscopy. 

**Statistics analysis**

The results are represented as mean±SEM and the data normality was checked using Kolmogorov–Smirnov test. Values were analyzed through SPSS ver.20 by one-way ANOVA followed by Tukey’s post-hoc test. A p<0.05 was regarded as significant difference.

## Results

**EEG power of experimental groups**

**Figure 2:**
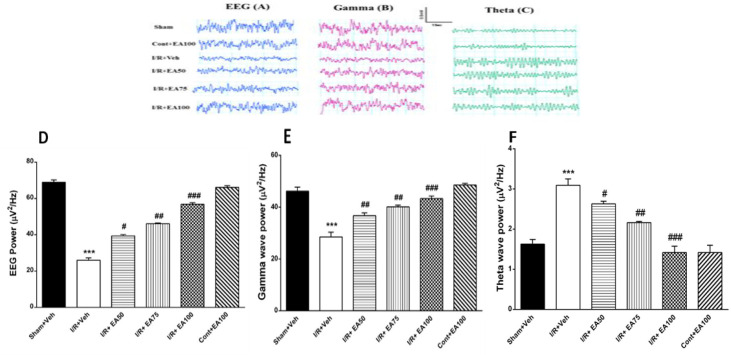
Effect of different doses of EA on brain hippocampal electrical activity recorded as local field EEG from CA1 area. Crude local EEG traces and electrical power (A and D), Gamma band traces and electrical power (B and E), theta band traces and electrical power (C and F) in different tested groups. Data are presented as Mean±SEM. (n=8). One-way ANOVA followed by Tukey’s post hoc test was used. Power of EEG and gamma band decreased in the I/R+Veh significantly compared to the sham+Veh (***p<0.001). Treatment with different doses of EA improved it dose-dependently compared to I/R+veh (#p<0.05, ##p<0.01 and ###p<0.001 respectively). Power of theta band was increased significantly in the I/R+veh compared to the sham+veh (***p<0.001) while administration of different doses of EA decreased it significantly (#p<0.05, ##p<0.01 and ###p<0.001 respectively). Sham+Veh; sham operated rats received DMSO/ normal saline (10%) as solvent of EA, I/R+Veh group; Ischemic rats received DMSO/ normal saline. I/R+EA; ischemic/reperfusion groups received 50, 75 or 100 mg/kg EA respectively. *** used to compare the I/R+Veh group with the sham+Veh. ## and ### used to compare the I/R+EA (50, 75 and 100 mg/kg,) groups with the I/R+Veh

Figure 2 illustrates the field local EEG traces recorded from CA1 area of the hippocampus in different groups with 8 rats. There were significant decreases of local EEG and gamma band electrical powers in the I/R+Veh group compared to the sham+Veh (p<0. 001, Figure 2A-C). Treatment with EA three times daily for 7 days increased significantly the local EEG in the I/R+EA50, I/R+EA75 and I/R+EA100 groups compared to I/R+Veh (p<0.05, p<0.01, p<0.001 respectively, Figure 2D), and gamma band power (p<0.01, p<0.001 respectively, Figure 2E) (F (5, 42) = 36.6, p<0.0001)). The theta band power was increased significantly in the I/R+Veh compared to the sham+Veh (F (5, 42) = 29.6, p<0. 001) and treatment with EA reduced it significantly in the I/R+50, I/R+75 and I/R+100 groups compared to I/R+Veh (p<0.05, p<0.01, p<0.001 respectively) (Figure 2 D-F).

**Behavioral results**

**The passive avoidance task (PAT)**

**Figure 3:**
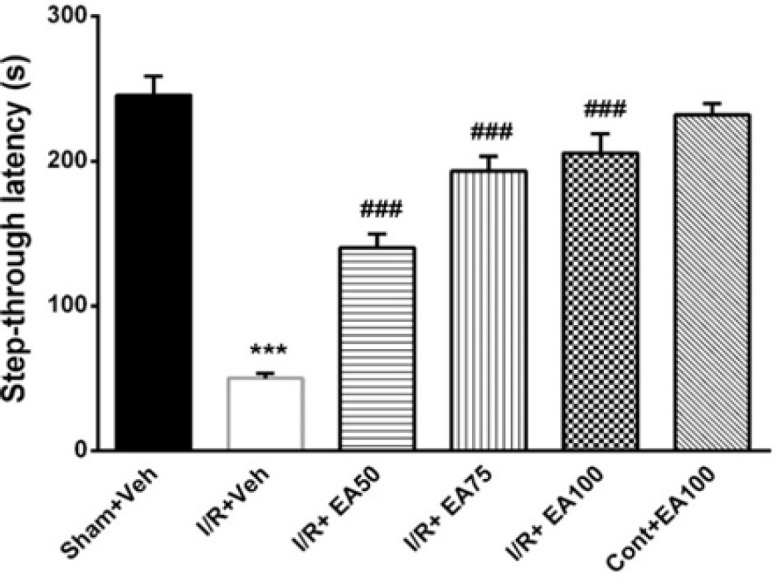
The step-through latency (STL) as the index of passive avoidance memory in different tested groups. Data are presented as Mean±SEM. (n=6), and were analyzed by one-way ANOVA followed by Tukey’s post hoc test. Pain threshold in the I/R+veh decreased significantly in compared to the sham+veh (p<0.001). Treatment of the ischemic group with different doses of EA returned it significantly compared to I/R+Veh (###p<0.001). Sham+Veh; sham operated rats received DMSO/ normal saline (10%) as solvent of EA, I/R+Veh group; received DMSO/ normal saline. I/R+EA; ischemic/reperfusion groups received 50, 75 or 100 mg/kg EA respectively three times daily for one week. *** used to compare the I/R+Veh groups with the sham+Veh. ### used to compare the I/R+EA (50, 75 and 100 mg/kg) groups with the I/R+Veh group

Figure 3 reveals the PAT in order to evaluate the passive avoidance memory in a shuttle box device. The initial latency (IL) had no difference between the experimental groups but the step-through latency (STL) as memory retention showed a signiﬁcant reduction in the I/R group compared to the Sham+Veh (p<0.001). Treatment with EA (50, 75 and 100 mg/kg, ip) improved it significantly compared to the I/R+Veh group (F (5, 30) = 51.55, p<0.001).

**Motor coordination**

**Figure 4:**
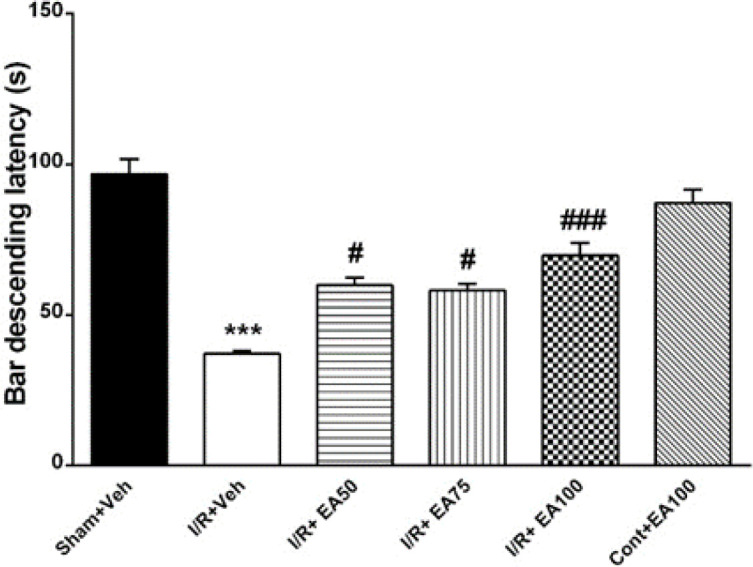
The effect of EA on the bar descending latency in rotarod test. Data are presented as Mean±SEM. (n=6). Data were analyzed by one-way ANOVA followed by Tukey’s post hoc test. Sham+Veh; sham operated rats received DMSO/normal saline (10%) as solvent of EA, I/R+Veh group; received DMSO/normal saline. Motor balance in I/R+veh group decreased significantly related to sham+veh (p<0.001) while administration of EA in ischemic rats improved it significantly (#p<0.05 and ###p<0.001). I/R+EA; ischemic/reperfusion groups received 50, 75 or 100 mg/kg EA respectively three times daily for one week. *** used to compare the I/R+Veh group with the sham+Veh. #, ## and ### used to compare the I/R+EA (50, 75 and 100 mg/kg) groups with the I/R+Veh group

Figure 4 shows the bar descending latency of rats on the rotarod device as an index for motor coordination and balance with 8 rats in each group. Bar descending latency was decreased in the I/R+Veh group significantly (p<0.001) compared to the Sham+Veh. Treatment with different doses of EA increased it significantly in the I/R+EA50 (p<0. 05), I/R+EA75 (p<0.05) and I/R+EA100 (p<0. 001) versus the I/R+Veh (F (5, 30) = 49.6, p<0.0001).

**Oxidative stress**

**Figure 5:**
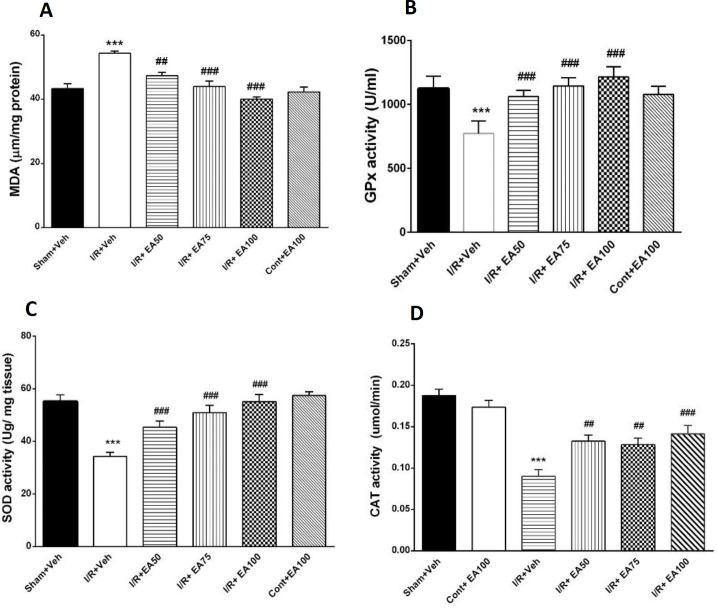
Effect of different doses of EA on brain MDA (A), GPx (B), SOD (C) and CAT (D) activities in different treatment groups. Data are presented as Mean±SEM. (n=6) and were analyzed by one-way ANOVA followed by Tukey’s post hoc test. MDA increased significantly in I/R+veh compared with sham+veh (p<0.001) and treatment the ischemic rats with different doses EA decreased MDA levels (p<0.001). Ischemia caused significant decreases in the antioxidant parameters in the sham+veh group (p<0.001) while treatment of the ischemic rats with different doses of EA restored them (p<0.001). Sham+Veh; sham operated rats received DMSO/ normal saline (10%) as solvent of EA, I/R+Veh group; received DMSO/ normal saline. I/R+EA; ischemic/reperfusion groups received 50, 75 or 100 mg/kg EA respectively three times daily for one week. *** used to compare the I/R+Veh groups with the sham+Veh. ## and ### used to compare the I/R+EA (50, 75 and 100 mg/kg) groups with the I/R+Veh

Figure 5 (A-D) presents the changes of brain MDA (A) and anti-oxidative GPx (B), SOD (C) and CAT (D) parameters in all tested groups with 8 rats in each group. There was a significant increase of MDA in the I/R+Veh group compared to the Sham+Veh (p<0.001). The antioxidant enzymes activity such as GPx (p<0.001), SOD (p<0.001) and CAT (p<0.001) were decreased in the I/R+Veh group compared to the Sham+Veh. Administration of EA decreased MDA in the treated groups I/R+EA50 (p<0.001), I/R+EA75 (p<0.001) and I/R+EA100 compared to I/R+veh (p<0.001), F (5, 30) =15.74, p<0.0001). The activity of antioxidant enzymes including GPx increased significantly (p<0.001, for I/R+EA50, 75 and 100, F (5, 30) = 51.5, p<0.0001)), SOD (p<0. 001, for I/R+EA50, 75 and 100, (F (5, 30) = 85.8, p<0.0001)) and CAT (p<0.01 for I/R+EA50, 75 and p<0.001, for I/R+EA100, (F (5, 36) = 25.5, p<0.0001)) compared to the I/R+Veh. 

**Histological verification**

**Figure 6:**
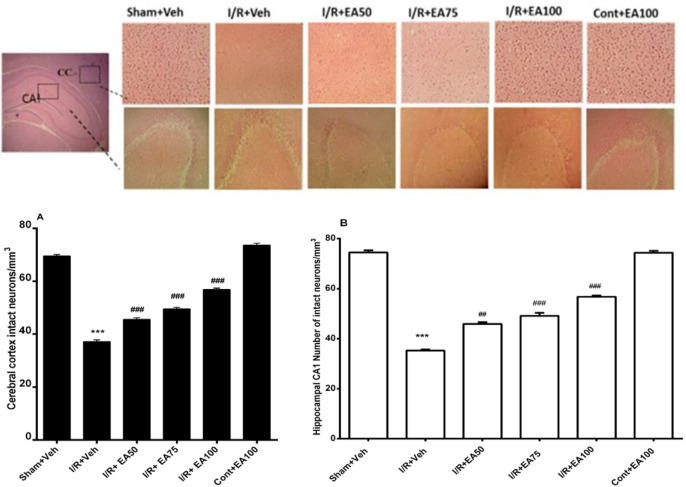
Histological changes in the cerebral cortex (CC) and hippocampal CA1 regions (H&E staining) of different groups. Data are presented as Mean±SEM. (n=8) and were analyzed by one-way ANOVA followed by Tukey’s post hoc test. Number of intact neurons in cerebral cortex (A) and hippocampal CA1 (B) of ischemic rats were decreased significantly in comparison to shame+veh group (p<0.001) while in treated groups with different doses of EA neuronal lose significantly reduced (p<0.001 in both brain regions). Sham+Veh; sham operated rats received DMSO/ normal saline (10%) as solvent of EA. I/R+Veh group; ischemic rats received DMSO/ normal saline. I/R+EA; ischemic groups received 50, 75 or 100 mg/kg EA respectively three times daily for one week. *** used to compare the I/R+Veh groups with the sham+Veh. ### used to compare the I/R+EA groups with the I/R+Veh

Figure 6 illustrates neuronal viability in the cerebral cortex (CC) and hippocampal CA1 region after H&E staining in different tested groups (n=8 in each group). Significant differences were shown in the neuronal viability in the CC (F (5, 42) =534.9, p<0.0001) and CA1 region (F (5, 42) =398.5, p<0.0001). Further analyses showed that the number of intact neurons was significantly lower in the I/R+Veh group than the Sham+Veh in both CC (p<0.0001) and CA1 region (p<0.0001). EA treatment at 50, 75 and 100 mg/kg doses significantly increased the number of intact neurons in CC (p<0.0001, p<0.0001, and p<0.0001 respectively) and CA1 region (p<0.0001, p<0.0001, and p<0.0001 respectively) compared to I/R+veh. No significant differences were observed among the different doses of EA.

## Discussion

The current study showed that cerebral ischemia could impair cognitive and motor behaviors due to brain oxidative damage. After I/R induction, the mean electrical power of gamma band in recorded local EEG from hippocampal CA1 area decreased significantly compared to the Sham group. The results of the present study also showed that administration of different doses of EA improved memory and motor balance while lowered MDA level, and increased the activity of antioxidant enzymes such as SOD, GPx, and CAT in the ischemic rats. 

In recent years, oxidative stress has been considered a predisposing factor for many diseases including brain ischemia and was also described as a mechanism involved in many neurological disorders. Increased ROS content can damage cellular components such as proteins, lipids, and nucleic acids. Excessive oxidative stress can even lead to apoptosis and necrosis (Rosenfeldt et al., 2013 ▶). On the other hand, lack of ROS metabolism regulation can severely affect the proper function of mitochondrial cells. In the literature, there are associations between increased mitochondrial ROS production and neuronal and endothelial damage (Widlansky and Gutterman, 2011 ▶), neurodegeneration (Orsucci et al., 2011 ▶), ischemic cell aging, and cerebral ischemia/ reperfusion (Chen et al., 2011 ▶).

Cerebral ischemia has been recognized as one of the most debilitating brain injuries. Cerebral ischemia results in elevated ROS content and activation of paths leading to cell death in vulnerable brain regions (Bhardwaj et al., 2013 ▶). During ischemia, reperfusion also can damage the brain tissue severely (Mitchell et al., 2017 ▶). This finding is consistent with current results. Blood supply can return oxygen to the cells and interrupt the release of free radicals, leading to programmed cell death (Liu et al., 1993 ▶). Glutamate intoxication and increased calcium level are key factors in the early stages of ischemic cell death (Moskowitz et al., 2010 ▶). 

Active oxygen and nitrogen species are naturally and constantly produced in the body and are removed and controlled by internal enzymes (SOD, GPx, and CAT, etc.). With an increase in the production of free radicals, the defense mechanism of the body is weakened, and macromolecules (e.g. proteins, lipids, and nucleic acids) are oxidized and destroyed (Oke et al., 2009 ▶). The brain tissue is rich in phospholipids which can be invaded by high levels of ROS to initiate lipid peroxidation (Labrousse et al., 2009 ▶). Lipid peroxidation results in the production of toxic aldehydes, among which MDA is known as one of the most toxic ones (Hong et al., 2004 ▶). 

Hippocampal cells which play an important role in memory formation, are highly vulnerable to cerebral ischemia. Cytokines produced in cerebral ischemia lead to the loss of pyramidal cells in the CA1 and CA3 regions of the hippocampus. Reperfusion also produces ROS and causes neuronal cell damage, therefore, memory impairment may be caused by increased inflammatory cytokines and ROS and decreased levels of enzymatic and non-enzymatic antioxidants in the hippocampus (Pan et al., 2017 ▶). Free radicals are naturally eliminated by intracellular antioxidant enzymes such as SOD, GPx, and CAT through the scavenging of free radicals (Kakita et al., 2002); so, the neuronal damage can be minimized (Calapai et al., 2000 ▶). 

Phenolic compounds have antioxidant properties and can prevent the deleterious effects of free radicals in the cells (Visioli et al., 2002 ▶). Flavonoids are also involved in scavenging free radicals by augmenting the body’s natural antioxidant enzymes such as SOD, GPx, and CAT (Zhang et al., 2013 ▶). In the current work, treatment of the ischemic rats with EA increased activity of the antioxidative enzymes as confirmed by other investigations. EA, like other phenolic compounds, exerts antioxidant effects which are caused by either counteracting the deleterious effects of oxidative stress or activating cellular antioxidant systems, such as SOD, CAT, and GPx (Han et al., 2006 ▶). 

Some evidence has shown that acetylcholinesterase inhibitors which increase acetylcholine in the synaptic cleft, improve cognitive function in rodents and humans, whereas anticholinergic drugs may impair cognition in different models (Zarrindast et al., 2002 ▶). It has been previously reported that EA improved learning and memory in mice with Alzheimer's disease through complex mechanisms such as antioxidant activity and inhibition of acetylcholinesterase in the brain (Tancheva et al., 2017 ▶). 

Cerebral ischemia is the leading cause of death and long-term disability worldwide. Among the most common disabilities, the motor disorders are the most common and debilitating ones which may affect the patient abilities to perform daily activities (Menezes et al., 2020 ▶). In the present work, administration of effective doses of EA (especially 75 and 100 mg/kg) was able to improve all behavioral parameters, brain electrical activity and oxidative stress in I/R rats. Since consumption of high doses of EA (>2000 mg) has few side effects (Bhandary et al., 2013 ▶), these results can offer a bright future for the recovery of patients with cerebral ischemia after adequate clinical trials.

The local field EEG signal is one of the electrical outputs of the brain, affected by the anatomical and physiological properties of the brain. These signals are produced as a result of the brain cell electrical function; therefore, brain structure and function affect the production of these signals (de Leon et al., 2001 ▶). Bezerianos et al. confirmed that there is a significant relationship between cerebral I/R and the relative electrical power of EEG signals (Bezerianos et al., 2003 ▶). Like our findings, numerous findings in other studies have established the role of cerebral ischemia in EEG power (Yu, et al., 2009 ▶). The direct EEG changes detected after stroke are a direct result of the decline of the cerebral blood flow that later results in neuronal impairment or neuronal death (Rabiller et al., 2015 ▶). 

Unfortunately, we have some limits in the present research so that we could not measure the changes of cerebral blood flow by laser Doppler device probe. It has been shown that theta and delta bands are sensitive to cerebral ischemia (Kearse et al., 1993 ▶). 

Evidence showed that the power of rhythmic slow activity recorded during freely moving behavior and the power of slow delta activity during alert immobility decreased monotonically. In addition, degenerating neurons were also observed in the hilus of the dentate gyrus (Buzsaki et al., 1989 ▶). 

EEG has been revealed as an unfailing tool for diagnosing dementia pathologies. The use of EEG in Alzheimer's disease has attracted wide interest (Tsolaki et al., 2014 ▶). Moretti et al. showed that theta/gamma ratio of relative power at peak frequency is linked with memory deterioration significantly (Moretti et al., 2009 ▶). Previous studies confirm our findings. It has been shown that the cerebral I/R could negatively affect the electrical activity of hippocampal neurons and thereby cognition (Ravindran, 2019 ▶). 

Several studies have shown that there is a noteworthy correlation between EEG changes and motor modifications, proving a link between them, even though a causal relationship still remains to be recognized (Bellesi et al., 2012 ▶). In addition, there is evidence that theta oscillations play a role in integrating sensorimotor information (Bast et al., 2003 ▶). Overall, EA may have a protective effect on the EEG pattern by activating antioxidant enzymes. This was proved by an earlier study (Hoseinynejad et al., 2017 ▶). 

Taken together, these results indicate that EA presents potential neuroprotection against neuronal damage via exerting antioxidative activity, thereby ameliorating I/R-induced motor and cognitive impairments in the rats, suggesting that EA may be a potential therapeutic agent against I/R. However, there were some financial and technical limitations for measuring the apoptosis and anti-apoptosis related proteins as well as enzymes related to BBB integration after cerebral ischemia. 

Cerebral ischemia leads to motor disorders, cognitive impairments, and brain tissue oxidative damage. Based on the current and our previous findings, EA is a strong antioxidant. It could improve BBB permeability, brain edema, prevent neuronal death and thereby restore hippocampal electrical activity in treated I/R rats. 

## Conflicts of interest

The authors have declared that there is no conflict of interest.
